# Expression, purification and analysis of the anti-HIV Cyanovirin-N produced in transgenic soybeans seeds

**DOI:** 10.1186/1753-6561-8-S4-P105

**Published:** 2014-10-01

**Authors:** Andre Murad, Nicolau Cunha, Cristiano Lacorte, Marly Coelho, Giovanni Vianna, Elibio Rech

**Affiliations:** 1 Embrapa Recursos Genéticos e Biotecnologia - Estação Parque Biológico, 70770-910, Brazilia, DF, Brazil

## Background

Human immunodeficiency virus (HIV) is infecting over 34.0 million people worldwide, being responsible for one of the major current pandemics [1]. The most severely affected region is the Sub-Saharan Africa where nearly 1 in 20 adults is infected with HIV, accounting for 69% of infections worldwide [1]. Several strategies to halt HIV spread are currently being pursued, including the use of ectopic microbiocides. This approach is particularly important for women, among which the infection rate can be almost three more times than men. Cyanovirin-N (CVN) is a lectin-like protein isolated from blue algae *Nostoc ellipsosporum*. CVN is highly thermo stable and is capable of binding to HIV GP120 protein blocking viral infection [2,3]. In this report we describe the use of transgenic soybean plants as a potential platform to achieve large scale, cost-effective production of CVN.

## Methods

The fragment of 306bp corresponding to CVN coding region was amplified by PCR from pET30b-CVN (BioSyn) vector and cloned into bombardment vector pbCong. This vector contains the promoter and the complete signal peptide of the soybean b-conglycinin gene with a CaMV35S terminator. The final vectors pbCongCVN and pAC321 (containing Imazapyr herbicide resistance gene) were co-bombarded in a 1:1 ratio into the apical meristem of somatic embryonic axes from mature soybean seeds cv. BR-16, utilizing a particle bombardment device [4]. Transgenic R0 plants were cultivated under green-house conditions to produce seed. R1 seeds were analyzed by PCR and ELISA-gp120 followed by nanoUPLC-MS^E ^[5] to characterize and quantify the expression levels of recombinant CVN. Seeds were grinded and proteins were extracted using 50mM Tris-HCl pH 8.0 in 1:20 (w:v) ratio. Total soluble proteins (TSP) were separated using gel filtration sephadex s200 followed by HPLC C4 reversed phase. Fraction containing semi-purified recombinant CVN were used for anti-HIV assay.

## Results and conclusions

From 1.000 embryonic axes used in bombardment, 20 plants were recovered from herbicide selection and only 8 contained CVN transgene. After ELISA-gp120 two plants showed CVN binding to gp120 HIV glicoprotein. NanoUPLC-MS^E ^results from TSP indicates a expression level of 1.5%. Structure characterization showed correct sequence which was possibly glycosilated. Anti-HIV assays from semi-purified SOY-CVN indicates microbicidal activity that was 10 folds less effective than the CVN control produced in *Escherichia coli*. Mass spectrometry quantification of this fraction confirms 10 fold dilution with other soybean seeds proteins, possibly due to a high affinity binding of CVN for glycinin and b-conglycinin which are abundant in soybean seeds. Therefore, production of pure recombinant CVN from soybean seeds is currently being optimized, and may contribute the development of an important HIV infection prevention method.
